# Hooves, Sensors, and Signals: Precision Approaches to Automated Lameness Detection in Dairy Cattle

**DOI:** 10.3390/ani16172643

**Published:** 2026-08-24

**Authors:** Chloe C. Hudson, Molly C. Nicodemus, Marcus M. McGee, Madeline G. McKnight, Kelsey M. Harvey

**Affiliations:** 1 Department of Animal and Dairy Sciences, Mississippi State University, Starkville, MS 39762, USA; mcn16@msstate.edu (M.C.N.); mm283@msstate.edu (M.M.M.); 2 Prairie Research Unit, Mississippi State University, Prairie, MS 39756, USA; mgm575@msstate.edu (M.G.M.); ks3114@msstate.edu (K.M.H.)

**Keywords:** artificial intelligence, automated lameness detection, computer vision, dairy cattle, precision livestock farming, sensor technology, welfare

## Abstract

Lameness remains one of the most significant welfare and economic challenges in modern dairy production. Traditional detection methods, particularly visual locomotion scoring, are limited by subjectivity, inconsistent application, and infrequent monitoring, which often delays the recognition of painful lesions. This review synthesizes recent validation studies of automated lameness detection (ALD) technologies and evaluates their diagnostic performance, validation design, and practical implementation across dairy systems. Studies were screened for relevance by a single reviewer based on title, abstract, and full-text content. Included studies represented sensor-based, pressure-based, vision-based, and multimodal detection platforms, with reported accuracies ranging from approximately 70% to 98% depending on modality and environmental setting. Vision-based systems demonstrated strong performance in controlled conditions, whereas field-validated systems showed more moderate but potentially more generalizable accuracy. Pressure-based platforms reported high diagnostic discrimination via area under the curve (AUC) analysis but face infrastructural limitations in commercial settings. Risk-of-bias assessment indicated that controlled experimental studies without external validation may overestimate deployment performance. Despite technological advances, variability in validation protocols, lesion thresholds, and environmental robustness limits direct comparison across systems. Future research should prioritize multi-farm external validation, standardized benchmarking frameworks, and multimodal integration within precision livestock farming ecosystems to improve reliability and adoption.

## Simple Summary

This manuscript reviews current technologies for automated lameness detection (ALD) in dairy cattle. Traditional visual locomotion scoring is subjective and inconsistent, often missing early signs of potentially painful causes of lameness. This delays treatment and causes major welfare and economic losses. This review summarizes findings from 43 studies evaluating various ALD systems, with reported accuracies ranging from 70 to 98% depending on the technology and environment used. Wearable sensors can detect early changes in activity and gait, whereas pressure systems provide detailed weight distribution data but require specialized infrastructure. Vision-based systems, including deep learning and three-dimensional approaches, show strong performance in controlled settings but are sensitive to varying environmental conditions. Collectively, future work should emphasize standardized benchmarking, multi-farm and environment validation, and integration of ALD tools within existing precision livestock farming systems to improve adoption and practicality.

## 1. Introduction

Lameness is a major health and welfare concern in dairy cattle and is associated with substantial economic losses due to treatment costs, reduced productivity, and reduced cow longevity [1,2,3]. Reported prevalence in commercial dairy herds is often estimated at approximately 20–30%, although values vary across production systems, geographic regions, housing and flooring conditions, herd management conditions, and detection methodologies [3,4]. Economic losses associated with individual lameness cases have been estimated at approximately USD $120–$300 due to reduced milk yield, impaired fertility, treatment expenses, and increased culling risk [1,2]. These values are based on studies published more than a decade ago and likely underestimate current costs, and more recent analyses may suggest higher economic impacts due to inflation, increased veterinary and labor expenses, and improved accounting of indirect production losses. Beyond its economic consequences, lameness represents a significant welfare concern, as it is strongly associated with chronic pain, altered gait, reduced mobility, and long-term declines in quality of life [5]. Animal welfare has also become an important determinant of consumer perception and industry acceptance, with increasing societal scrutiny of livestock production practices [6,7]. Consequently, the prevalence of conditions such as lameness has implications not only for the animal, but also for public trust, market dynamics, and the long-term sustainability of dairy systems [8,9].

Conventional detection of lameness in dairy cattle relies primarily on visual locomotion scoring, routine producer observation, and periodic assessments by hoof trimmers or veterinarians [10,11,12]. Widely used locomotion scoring systems, including the five-point scale described by Sprecher et al. [13], classify cows based on observable gait and posture characteristics such as back arch, stride length, asymmetry, and altered weight-bearing [10,12]. These systems provide a structured and practical approach for identifying mobility impairment; however, their effectiveness is limited by observer subjectivity, inconsistent application, and variable agreement among evaluators [12,14,15]. For example, Thomsen et al. [16] reported weighted interobserver kappa values ranging from 0.24 to 0.68, with mean values of 0.48 before and 0.52 after observer training, demonstrating only moderate interobserver agreement in visual lameness scoring.

Because traditional assessment methods are visual, intermittent, and dependent on human observation, early gait or behavioral abnormalities may remain undetected until lesions progress to more clinically obvious stages [12,17]. This limitation is especially relevant in large herds, where producers may have limited time to observe individual cows and subtle locomotor changes can be missed during routine management activities [9,10,11,12]. Delayed recognition can allow pathology to progress, increasing treatment costs, prolonging recovery, and raising the risk of chronic impairment or premature culling [1,12]. Collectively, these limitations highlight the need for more objective, repeatable, continuous, and scalable approaches to lameness detection.

These limitations have driven increased interest in automated lameness detection (ALD) systems that utilize wearable sensors, including inertial measurement units (IMUs), pressure-sensing platforms, and computer vision algorithms integrated with machine learning (ML) models to provide objective, continuous, and potentially earlier identification of mobility impairments [11,18,19]. Within the broader framework of precision livestock farming (PLF), such technologies have the potential to enhance management decision-making, improve animal welfare outcomes, and optimize production efficiency through data-driven monitoring systems [6,20,21]. However, reported diagnostic performance varies widely across sensing modalities, validation protocols, and production environments. Differences in reference standards, lameness thresholds, and study design also complicate direct comparisons among systems [22]. A structured evaluation of existing validation studies is therefore necessary to assess both diagnostic accuracy and real-world applicability.

## 2. Scope and Organization of the Review

Accordingly, this targeted review synthesizes current research on ALD technologies in dairy cattle, with emphasis on diagnostic performance, validation approaches, and practical implementation within commercial systems. The review primarily focused on studies published within the last decade; however, earlier foundational studies were included when they provided important background on the development of ALD methods. A structured literature search was conducted from August 2025 to January 2026 in Web of Science, Scopus, and PubMed. These databases were selected because they provide broad coverage of peer-reviewed literature in animal science, veterinary medicine, agricultural technology, and biomedical research. Search terms included combinations of “automated lameness detection,” “dairy cattle,” “dairy cows,” “wearable sensors,” “computer vision,” “machine learning,” “locomotion scoring,” and “sensor-based monitoring.” Search terms were modified slightly across databases to account for differences in database indexing, search syntax, and controlled vocabulary while maintaining the same overall search concepts. Studies were screened for relevance by a single reviewer based on title, abstract, and full-text content. Because screening was conducted by a single reviewer, the possibility of selection bias cannot be fully excluded; however, studies were selected using predefined relevance criteria focused on ALD technologies in dairy cattle. The initial database search returned 300 records, of which 150 remained after removal of duplicates. Following relevance-based screening, 43 studies were included and evaluated to provide an overview of current advancements, diagnostic approaches, implementation challenges, and remaining gaps in ALD development (Supplementary Table S1). Because this review does not follow full PRISMA guidelines, a complete systematic flow diagram is not provided; however, the above summary reflects the major steps of the screening funnel. Additional references included in this review are background, methodological, or mechanistic citations used to support interpretation.

The present review is organized to progress from foundational sensing technologies to advanced integrative approaches. This review first addresses sensor- and force-based systems, including wearable sensor technologies, force-based pressure systems, and physiological or behavioral indicators. It then examines vision- and image-based approaches, including two-dimensional (2D) deep learning methods and three-dimensional (3D) reconstruction techniques. Building on these modalities, the review explores hybrid and multimodal systems, including data fusion and multimodal integration, machine learning-based modeling, and key technological implementation challenges. We evaluate applications under on-farm conditions, including environmental and operational constraints, integration within PLF systems, and current commercial technologies. Finally, we address the benefits of automation, including welfare and detection advantages, production outcomes, and system-level considerations, before concluding with current limitations and future perspectives.

## 3. Automated Lameness Detection Technologies

**Figure 1:**
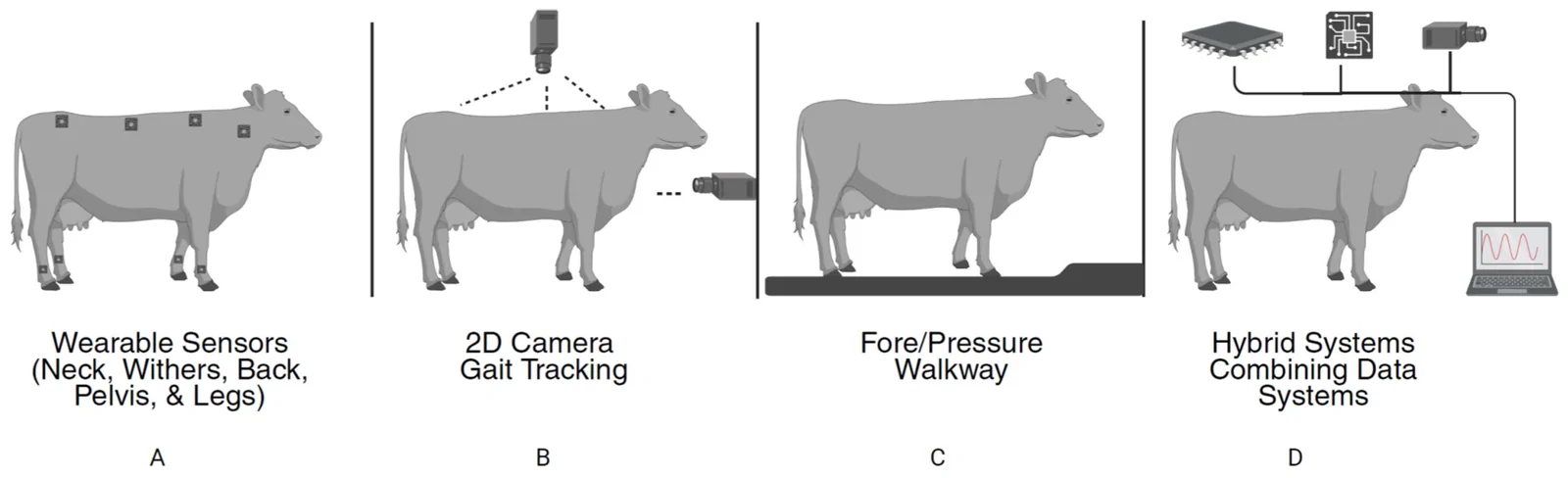
Representative automated lameness detection (ALD) modalities in commercial dairy systems. Schematic illustrations depict common sensing approaches, including (**A**) wearable sensor systems such as accelerometers and inertial measurement units (IMUs) for continuous activity and stride monitoring, (**B**) overhead two-dimensional (2D) and three-dimensional (3D) computer vision systems for kinematic gait tracking and posture analysis, (**C**) force or pressure plate walkways for quantifying ground reaction forces and stance asymmetry, and (**D**) hybrid multimodal systems integrating sensor, vision, and behavioral data streams. These modalities and their applications are based on previously reported ALD studies [6,11,12,23]. Created by the authors using BioRender.com (2026). Software was selected for figure-specific functionality.

Automated lameness detection systems have emerged as scalable, data-driven tools designed to overcome the subjectivity and episodic limitations of traditional visual locomotion assessment [6,11,12]. By enabling continuous monitoring of locomotor and behavioral parameters, these systems provide the potential for earlier detection of mobility impairments and more timely intervention [6,11,23]. Contemporary ALD platforms employ a range of sensing modalities, including wearable biomechanical sensors, floor-based force or pressure measurement systems, computer vision technologies, and integrated multimodal monitoring approaches [11,24]. Representative implementations of these sensing modalities in commercial dairy systems are illustrated in Figure 1. Advances in machine learning and deep neural network architectures have further enhanced signal interpretation and classification accuracy, enabling detection of subtle locomotor deviations that may precede clinically observable lameness [19,25]. However, stratified performance metrics across multiple pre-clinical days remain largely absent from the literature, representing an important area for future benchmarking.

### 3.1. Sensor- and Force-Based Systems

Sensor-based ALD platforms utilize wearable or environmental devices to quantify biomechanical and behavioral indicators of impaired mobility [11,24]. These systems continuously collect movement and activity data, which are processed using statistical or machine learning algorithms to identify deviations from normal locomotion patterns [19,25,26]. As a core component of PLF, sensor-based technologies support continuous health surveillance and management in modern dairy systems [6,27]. However, despite their potential for continuous and objective monitoring, sensor-based ALD systems face several limitations related to data variability, infrastructure requirements, and scalability in commercial dairy environments [11,23,24,28].

#### 3.1.1. Wearable Sensor Systems

**Figure 2:**
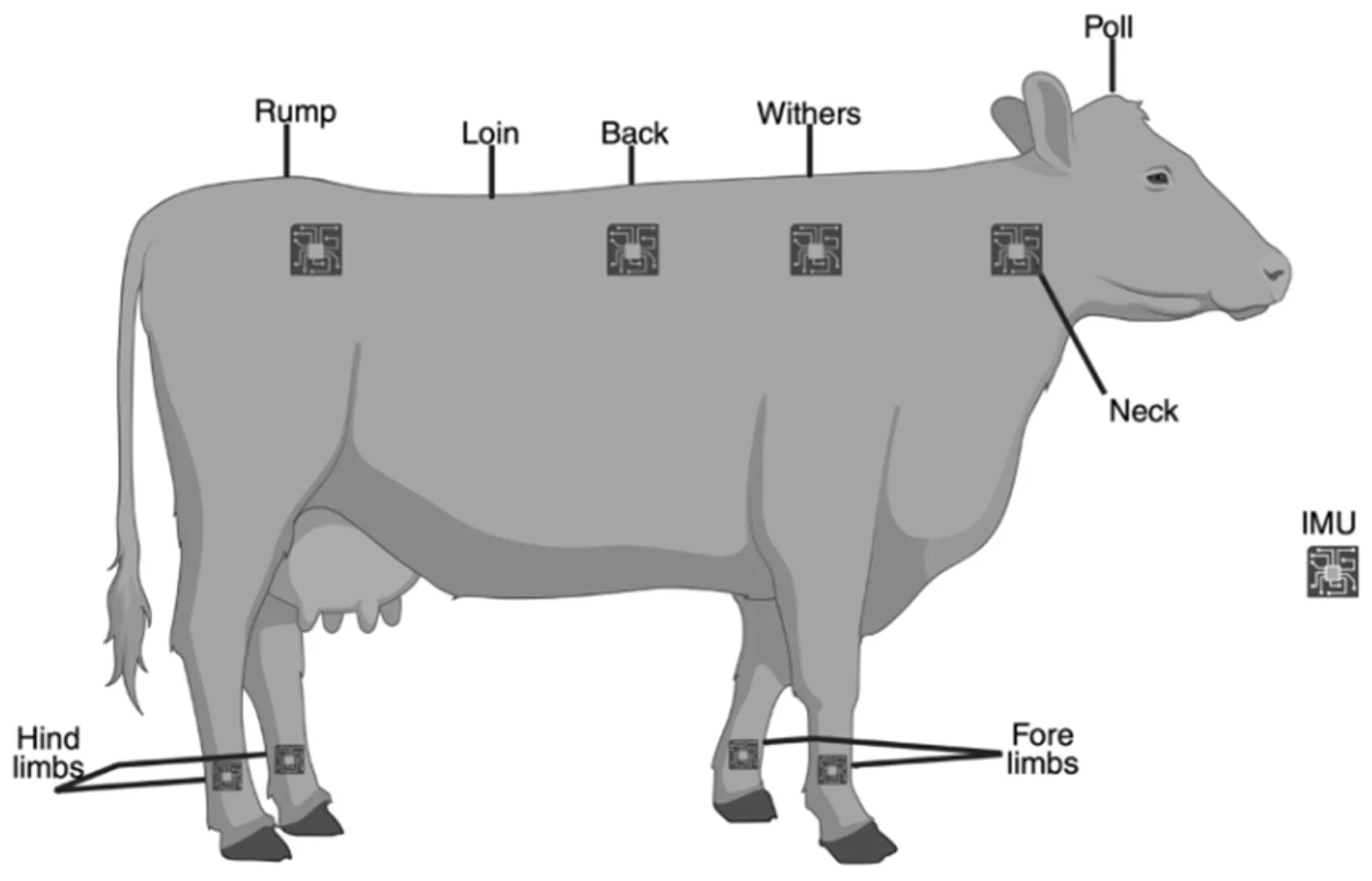
Representative anatomical placement sites for inertial measurement units (IMUs) used in automated gait analysis of dairy cattle. Sensors are positioned at axial landmarks (poll, withers, back, loin, sacrum) and distal limb segments to capture trunk motion and stride asymmetry. Placement locations and biomechanical variables are based on previously reported methodologies in automated lameness detection (ALD) studies [3,17,23,24,29]. Created by the authors using BioRender.com (2026). Software was selected for figure-specific functionality.

Wearable systems, including tri-axial accelerometers, pedometers, gyroscopes, and IMUs, are commonly attached to the distal limb, collar, ear tag, trunk, or other anatomical locations to record locomotion and behavioral parameters such as stride frequency, step asymmetry, activity levels, lying duration, and lying-standing transitions [29,30,31,32,33]. These systems are designed to detect deviations from normal movement patterns, including reduced stride length, increased recumbency time, altered activity, and changes in weight-shifting behavior, which may occur during early-stage lameness or before overt clinical signs become visually apparent [29,33,34,35]. Sensor placement at anatomical landmarks such as the sacrum, withers, poll, or distal limbs enables measurement of both limb kinematics and trunk dynamics, allowing wearable systems to capture different dimensions of locomotor impairment [17,24]. Despite strong diagnostic performance, wearable ALD systems face several practical disadvantages in commercial dairy environments. Mounting stability remains a major challenge, as devices attached to limbs, collars, or axial landmarks are prone to self-removal, loosening, or breakage due to rubbing, grooming, environmental contact, or interactions with other cows. Representative anatomical placement sites for inertial measurement units used in automated gait analysis are illustrated in Figure 2.

Several studies support the use of wearable and activity-based monitoring for lameness detection. Mazrier et al. [31] investigated pedometric activity as an early indicator of lameness and used a 5% reduction in activity relative to each cow’s previous 10-day average as a screening threshold. Among 38 lameness cases identified by reduced pedometric activity or clinical observation, 21 cows showed a reduction in activity from 7 to 10 days before clinical signs appeared, and 92% of cows identified by pedometric activity had activity decreases greater than 15% [31]. These findings suggest that activity monitoring may identify mobility changes before lameness becomes visually apparent [31].

Lying behavior has also been evaluated as an automated indicator of impaired mobility. Blackie et al. [34] used leg-mounted activity sensors to assess lying behavior in 59 lactating Holstein cows and found that lame cows spent significantly more time lying down than non-lame cows, averaging 13.0 h/day compared with 10.9 h/day. Similarly, Ito et al. [30] analyzed lying behavior in 1319 cows across 28 farms and reported that cows with severe lameness in deep-bedded stalls spent 12.8 h/day lying down compared with 11.2 h/day for cows that were not severely lame. In that study, cows lying more than 14.5 h/day had 16.2 times higher odds (95% confidence interval: 5.8 to 45.2) of being severely lame, while cows with lying bouts longer than 90 min had 3.0 times higher odds (95% confidence interval: 1.2 to 7.4) of severe lameness [30]. Together, these studies indicate that increased lying time and altered lying-bout patterns can provide useful behavioral indicators of lameness, although responses may vary with housing conditions [30,34].

Wearable systems are particularly attractive because they are scalable, non-invasive, and compatible with existing herd management platforms [6,11,27]. Recent work has focused on improving online detection and early-stage classification using wearable sensor data. Dai et al. [36] developed an online early lameness detection model using wearable IMU acceleration data and reported 80.6% health-status detection accuracy with a single-decision latency of 0.8 ms. The model achieved 89.11% recall and an 88.93% F1 score for lameness detection, supporting the potential of wearable sensor-based gait analysis for real-time monitoring and early intervention on large-scale farms [36].

Compared with more infrastructure-intensive modalities, wearable sensors currently represent one of the most practical approaches for large-scale on-farm implementation because they can continuously monitor individual cows without requiring specialized flooring or fixed imaging setups [6,11,23,27,36]. However, reliability remains influenced by sensor placement consistency, calibration, signal drift, and long-term data quality, indicating that continued refinement is needed to improve robustness and repeatability across commercial dairy environments [6,11,23].

#### 3.1.2. Force-Based Pressure Systems

Floor-based force and pressure measurement systems, including pressure-sensitive walkways, force plates, and instrumented alleys, are used to measure limb loading patterns, weight distribution, and pressure profiles during locomotion [10,17,24,37]. These systems provide objective kinetic measurements that can detect how cows redistribute body weight across limbs when locomotion is impaired [19,24,37]. Lame dairy cows typically exhibit reduced limb loading on the affected limb, altered pressure distribution, increased stance asymmetry, and changes in temporal gait characteristics [3,10,17,24,37]. Because these changes are directly related to weight-bearing and compensatory movement, force- and pressure-based platforms may support earlier identification of mobility impairment and provide quantitative data for evaluating lameness severity [17,37,38].

Several studies support the use of limb-loading and weight-distribution measures for objective lameness assessment [17,37]. Neveux et al. [37] showed that cows exposed to hoof discomfort reduced weight bearing on the affected hoof and redistributed most of that weight to the contralateral limb, demonstrating the biomechanical basis for using asymmetrical weight distribution as an indicator of lameness. Pastell et al. [17] further evaluated weight-distribution measures in 61 lactating dairy cows using limb weight ratio and hoof lesion data. Limb weight ratio was strongly associated with locomotion score in cows with sole ulcers, with an R^2^ of 0.79, and distinguished cows with sole ulcers from sound cows with no hoof lesions with an AUC of 0.87 [17]. The same approach also differentiated lame from non-lame cows with AUC values of 0.71 and 0.88, depending on the locomotion score threshold used [17]. These findings support the use of limb-loading asymmetry and weight-shifting behavior as key biomechanical indicators of lameness [17,37].

By quantifying limb loading and pressure distribution, force- and pressure-based systems demonstrate strong discriminative capacity and are often considered reference tools for biomechanical gait assessment [12,17,38]. However, their reliance on specialized walkways, controlled passage conditions, fixed installation sites, and higher infrastructure costs limits their practical application in commercial dairy environments [11,12]. Consequently, while these systems provide highly detailed kinetic information, they are often better suited for research, validation, or targeted monitoring settings than routine large-scale on-farm implementation [6,11,12].

#### 3.1.3. Physiological and Behavioral Integration

Emerging approaches incorporate physiological and behavioral indicators into ALD frameworks to complement locomotion-based detection [6,24,33,39,40]. Accordingly, this combination has the potential to improve detection sensitivity and robustness, particularly in environments where locomotion signals alone may be insufficient or confounded by external factors [33,41,42]. Sensors monitoring rumination, feeding behavior, and activity patterns have been explored as indirect indicators of lameness, given established associations between mobility disorders, reduced rumination, and altered feeding behavior [32,35,43,44,45,46]. By incorporating multiple data streams, these approaches may support earlier identification of lameness and improve classification performance across variable on-farm conditions [33,41,42].

However, physiological and behavioral indicators remain influenced by multiple factors, including nutrition, environmental conditions, and management practices, which may limit their specificity and reliability as standalone detection tools [6,11,23]. Consequently, these measures are best considered complementary inputs that enhance detection performance when integrated with primary locomotion-based systems rather than independent diagnostic indicators [24,33]. From a practical perspective, integration of physiological and behavioral monitoring within wearable sensor platforms offers a feasible pathway for commercial implementation due to scalability and compatibility with existing herd management systems [11,27]. As a result, these integrated sensor-based approaches may support wider adoption of multimodal ALD systems, although optimization of data quality and system reliability remains a key priority [6,11,23,27].

#### 3.1.4. Technological and Implementation Challenges

Sensor- and force-based ALD systems remain limited by several modality-specific challenges. Wearable sensors are influenced by placement consistency, calibration requirements, attachment stability, signal drift, and long-term data quality, all of which can affect reliability during continuous monitoring [6,11,23]. Force- and pressure-based systems provide detailed biomechanical information but require specialized infrastructure, controlled cow flow, fixed installation sites, and regular maintenance, which may limit their feasibility for routine on-farm use [6,11,12]. Physiological and behavioral indicators, including activity, rumination, feeding behavior, and metabolic measures, are also affected by non-lameness factors such as nutrition, lactation stage, housing, management practices, and environmental conditions, reducing their specificity when used as standalone detection tools [3,11,23]. Therefore, these approaches are most effective when interpreted as complementary components within broader ALD frameworks rather than as independent diagnostic systems [6,11,23].

### 3.2. Vision- and Image-Based Detection Systems

Computer vision technologies represent one of the most rapidly expanding areas of ALD research [12,38,47,48,49,50]. These systems capture continuous video data using fixed cameras and apply machine learning algorithms to extract locomotion features associated with gait asymmetry and postural deviations [19,26,51]. Cameras positioned in walkways, alleys, or barn environments enable non-invasive monitoring without the need for wearable devices or specialized flooring infrastructure [12,38,43,48,52]. Collectively, these characteristics position computer vision systems as a scalable and non-invasive approach for continuous, automated lameness monitoring in commercial dairy environments [38,48].

#### 3.2.1. Two-Dimensional and Deep Learning Systems

Conventional 2D systems quantify locomotion features such as back posture, walking speed, stride length, limb trajectories, and joint angles to classify lameness severity [38,52,53,54,55]. For example, Narli et al. [54] used computer vision analysis of back shape characteristics for automated lameness detection in dairy cattle, demonstrating the continued relevance of dorsal-line and postural features within vision-based ALD systems. More recent pose-estimation approaches have expanded the ability of 2D systems to quantify anatomical landmarks and movement patterns from video data. Modern vision pipelines commonly employ convolutional neural network (CNN)-based object detection architectures, including YOLO-family models and region-based approaches such as Mask R-CNN, to localize individual cattle or anatomical features before tracking, pose estimation, or temporal modeling is applied [50,56].

Barney et al. [56] developed a fully automated, multiple-cow, real-time lameness detection system using deep learning-based cattle detection and pose estimation. Their pipeline used a modified Mask R-CNN to estimate anatomical key points, followed by SORT to track individual cows across video frames and CatBoost to classify lameness from extracted gait and posture features [56]. The system evaluated features related to back arching and head position and achieved 100% accuracy for lameness detection and 94% accuracy for lameness severity classification using threefold cross-validation [56]. It should be noted that these results were obtained under controlled conditions using internal cross-validation. Taghavi et al. [55] further demonstrated the use of deep learning-based key-point detection in a practical indoor dairy setting by training the T-LEAP model to detect 17 anatomical key points from side-view video footage of Holstein-Friesian cows exiting the milking parlor. The model achieved 89% correctly detected key points across all landmarks on the test set, although back key-point detection was less consistent, emphasizing the importance of post-processing steps before key points can be translated into reliable gait features [55].

Early computer vision approaches relied on manually engineered features, whereas more recent deep learning frameworks enable automated feature extraction from raw video, improving detection of subtle gait asymmetries and temporal locomotion patterns [11,19,49,53,54,55,56]. Russello et al. [57] further demonstrated this approach by combining pose estimation with bidirectional long short-term memory networks, a type of recurrent neural network designed to analyze sequential data, allowing temporal gait information to be incorporated into lameness detection models. In addition, Szyc et al. [49] evaluated video-based ALD in dairy cows, further supporting the growing role of DL-driven video analysis within vision-based ALD systems. Collectively, these studies suggest that 2D vision systems are progressing from static image-based assessment toward more automated, landmark-based, multi-animal, and temporally informed gait analysis [49,55,56,57].

#### 3.2.2. Three-Dimensional Vision Systems

Three-dimensional vision systems employ stereo cameras, depth sensors, or structured-light imaging technologies to reconstruct the animal’s body during locomotion [38,52,53,58,59,60]. Unlike conventional 2D approaches, which primarily rely on visible image features from a single imaging plane, 3D systems capture depth information that allows spatial relationships among body regions to be quantified more accurately [58,61]. This enables more detailed assessment of locomotion-related features such as spinal curvature, back posture, limb displacement, stride dynamics, and weight-shifting patterns [38,52,53,59,60,61]. Because lameness often alters both limb movement and compensatory body posture, 3D reconstruction may improve detection of subtle gait abnormalities that are difficult to characterize using 2D image analysis alone [38,52,53,59,60,61].

For example, Abdul Jabbar et al. [58] demonstrated the potential of 3D depth-video analysis for early, non-intrusive lameness detection by using an overhead depth camera to record cows walking freely after milking. Their system automatically tracked anatomical regions such as the hooks and spine and used hip-height variation as a proxy for gait asymmetry. Using a linear support vector machine model, the approach identified the earliest lameness threshold with 95.7% accuracy, 100% sensitivity, and 75% specificity [58]. More recently, Bradtmueller et al. [61] used kinematic 3D coordinate data and machine learning to predict dairy cow locomotion ability, reporting 0.96 accuracy, precision, recall, and F1 score for the best-performing long short-term memory model after data augmentation and normalization [61]. Together, these studies support the value of three-dimensional spatial information for quantifying gait asymmetry, body movement, and locomotion impairment in dairy cows [58,61].

Depth-based systems may be particularly useful for measuring dorsal body curvature and asymmetry during walking, as changes in back arch and pelvic movement are commonly associated with altered weight-bearing and impaired locomotion [38,52,53,58,59,60]. By capturing the animal’s body surface in 3D, these approaches can reduce some limitations of 2D imaging, including perspective distortion and incomplete representation of body movement. As a result, 3D vision systems may provide improved spatial accuracy and greater sensitivity for detecting biomechanical changes associated with lameness [38,52,53,59,60,61].

Although 3D vision systems offer improved precision and richer biomechanical information, their practical use in commercial dairy settings requires careful consideration of system complexity [6,12]. Compared with 2D approaches, these systems generally require more complex camera placement, calibration, data processing, and computational resources, particularly when real-time analysis is needed [6,12,23]. Higher installation costs and technical requirements may therefore limit adoption in some commercial settings, especially unless systems are simplified, standardized, and validated under on-farm conditions [6,12,23].

#### 3.2.3. Infrared Thermography and Complementary Imaging Approaches

Another non-invasive imaging approach used in ALD systems is infrared thermography (IRT), which identifies localized increases in surface temperature that may reflect inflammation in the hoof or distal limb [18,29,62,63,64]. Because inflammatory processes increase local blood flow, thermographic imaging can identify temperature asymmetries that may precede visible locomotion abnormalities [63,64,65,66,67].

However, surface temperature is influenced by multiple physiological and environmental factors, limiting diagnostic specificity when thermography is used alone [6,18,29,63,68]. For example, variations in ambient temperature, humidity, and airflow can affect thermal readings, while physiological factors such as stress, activity level, stage of lactation, and systemic health status may also alter peripheral blood flow and surface temperature independent of lameness [6,12,18,23,29,68]. As a result, thermography is unlikely to function effectively as a standalone detection system and is better suited as a complementary modality within multimodal detection frameworks [11,12,23].

For example, Alsaaod and Büscher [18] evaluated digital IRT for detecting hoof lesions in dairy cows by measuring coronary band temperatures before and after claw trimming [18]. They found that claws with lesions had significantly higher coronary band temperatures than non-lesion claws and reported sensitivities of 80.0% to 85.7% using thermal threshold values [18]. However, hoof temperature was also strongly associated with ambient temperature, highlighting the need to control for environmental conditions when applying IRT in lameness detection [18].

Stokes et al. [64] further evaluated IRT as a rapid screening tool for dairy cattle foot lesions, with particular emphasis on digital dermatitis detection [64]. Although temperatures did not differ significantly between feet with digital dermatitis and those with other skin or claw horn lesions, the authors found that a 27 °C threshold for dirty feet correctly identified 80% of feet with lesions and 73% of feet without lesions [64]. These findings suggest that IRT may be useful for broad lesion screening, but its ability to distinguish specific lesion types is limited [64].

#### 3.2.4. Technological and Implementation Challenges

Vision-based ALD systems remain sensitive to environmental and operational conditions, including lighting variability, occlusion, coat contamination, animal positioning, walking speed, and crowding, all of which can reduce accuracy and generalizability in commercial settings [6,11,12,23,52]. Modality-specific limitations also remain important. Two-dimensional systems may be affected by perspective distortion and incomplete representation of body movement, whereas 3D systems require more complex camera placement, calibration, depth-data processing, and computational resources, which may limit scalability and real-time implementation [6,11,12,23]. Complementary imaging modalities such as IRT may improve detection of inflammatory or lesion-associated changes but lack diagnostic specificity when used independently, as thermal patterns can be influenced by environmental and physiological factors unrelated to lameness [6,11,12,23]. Therefore, although vision-based approaches offer strong potential for non-invasive lameness monitoring, continued validation under on-farm conditions is needed to improve robustness, standardization, and practical adoption [6,11,12,23,52].

### 3.3. Hybrid and Multimodal Systems

Hybrid ALD systems integrate multiple sensing modalities, including video analysis, wearable sensors, force measurements, and physiological monitoring, to improve diagnostic robustness and detection sensitivity [6,11,12,23,24]. These systems combine heterogeneous data streams and apply machine learning algorithms to identify patterns of locomotor impairment across multiple domains, including biomechanics, behavior, and physiology [19,30]. By capturing complementary information, multimodal frameworks provide a more comprehensive characterization of mobility abnormalities than single-modality approaches and improve resilience to environmental variability [6,23,45,69]. Table 1 summarizes the relative detection capabilities of major ALD modalities across lesion types and clinical presentations.

#### 3.3.1. Data Fusion and Multimodal Integration

Data fusion is a central component of multimodal ALD systems because it allows kinetic, kinematic, behavioral, and physiological signals to be evaluated simultaneously across multiple dimensions of locomotion and health [6,24,33]. Rather than relying on a single indicator of impaired mobility, multimodal systems combine complementary information from different data streams to improve detection sensitivity and robustness. For example, accelerometer-derived activity metrics can be combined with computer vision-derived gait parameters to capture both biomechanical alterations and associated behavioral changes [23,24,52,59]. Similarly, data from automated milking systems, behavioral sensors, body condition measures, and physiological indicators may be integrated to improve identification of mild or early-stage lameness that may not be consistently detected using locomotion variables alone [41,70,71,72].

Data integration can occur at multiple levels, including feature-level fusion and decision-level fusion [19,30]. In feature-level fusion, extracted variables from multiple modalities are combined into a unified dataset before model training, allowing the algorithm to identify relationships across locomotion, behavior, and physiological domains [19,30]. In decision-level fusion, outputs from independent models are aggregated to improve classification performance and reduce the influence of noise or modality-specific errors [19,30]. Feature-level approaches may enhance detection sensitivity by capturing complementary patterns across datasets, whereas decision-level frameworks may improve robustness by combining independent predictions from multiple detection streams [19,30].

Several recent studies demonstrate the value of integrating multiple data sources for ALD [41,70,71]. Dhaliwal et al. [70] introduced a bimodal AI framework that combined facial biometric data with accelerometer-derived movement metrics, using DenseNet-121 for image analysis, long short-term memory networks for time-series data, and a multi-head attention mechanism to fuse visual and locomotion features. In a 21-day study of six Holstein cows, the multimodal model achieved 99.55% accuracy and outperformed single-modality baselines, supporting the potential value of combined visual and movement-based monitoring for early lameness detection [70]. Small sample sizes, controlled environments, and lack of external validation substantially increase the risk of overestimating real-world performance. These limitations are consistent with broader evidence that internally validated or single-farm studies tend to report inflated accuracy compared with multi-farm, externally validated designs.

Džermeikaitė et al. [41] further demonstrated the value of multimodal integration by combining behavioral, physiological, blood biochemical, and milk composition parameters from 272 early-lactation dairy cows. Their random forest model achieved 97.04% validation accuracy, 100% specificity, and a normalized Matthews correlation coefficient of 0.94, while also identifying biologically relevant indicators associated with lameness, including elevated non-esterified fatty acids and body temperature, reduced water intake, milk protein, and milk lactose content [41].

In contrast, Lemmens et al. [71] evaluated a more production-oriented approach by combining routinely available sensor, automated milking system, animal, and farm-level data across ten dairy farms. Their best-performing random forest model achieved 0.75 accuracy, 0.72 sensitivity, and 0.78 specificity, suggesting that routinely collected production data may support practical detection of mild lameness, although performance may be more moderate under real-world conditions [71]. Together, these studies suggest that multimodal data fusion can expand ALD systems beyond isolated gait assessment toward more comprehensive and biologically interpretable production-level decision-support tools [41,70,71].

#### 3.3.2. Machine Learning and Multimodal Modeling

The integration of multiple data streams in ALD systems has necessitated the use of advanced analytical approaches capable of handling complex, high-dimensional datasets [19,25,30]. Advances in artificial intelligence (AI) have enabled sophisticated multimodal frameworks capable of processing heterogeneous sensor inputs simultaneously [19,25,30,73,74]. Machine learning models, including ensemble and deep learning approaches, improve predictive performance by capturing complex spatial and temporal patterns across datasets [72,75,76,77,78]. Multimodal systems combining physiological, behavioral, and locomotion data have demonstrated promising performance in large-scale datasets [38,41,42,71]. However, issues related to model generalizability, interpretability, and overfitting remain important considerations for real-world deployment [19,22,25,30].

#### 3.3.3. Physiological and Behavioral Augmentation

Physiological and behavioral monitoring provides an additional layer of information that complements locomotion-based indicators in multimodal ALD systems [6,24,33]. These indicators are valuable because lameness affects not only gait mechanics, but also activity, feeding behavior, rumination, weight distribution, metabolic status, and stress-related biomarkers [39,40,67,79,80,81,82]. For example, Chapinal et al. [40] evaluated gait, automated weight-distribution measures, lying behavior, step frequency, and walking speed in 57 lactating dairy cows, including 28 lame cows. Lame cows shifted weight between contralateral legs more frequently than non-lame cows, had greater rear-leg weight asymmetry, longer lying bouts, and slower walking speeds; variability in rear-leg weight distribution was the most accurate lameness predictor, with an area under the curve of 0.71 [40]. These findings suggest that automated measures of weight shifting and activity may help detect lameness and evaluate pain-related responses [40].

Behavioral monitoring has also been used to identify lameness-related changes in feeding and rumination patterns. Antanaitis et al. [39] used a noseband sensor to evaluate ingestive behavior in 20 fresh dairy cows and reported that healthy cows had rumination times 2.19 times higher than lame cows on the day of lameness diagnosis [39]. Lame cows also showed changes in eating time, drinking time, rumination chews, bolus count, and chewing patterns around the time of diagnosis, supporting the potential value of feeding-related sensor outputs as indirect indicators of mobility impairment and discomfort [39].

Physiological and biochemical indicators may provide additional insight into the systemic effects of lameness [67,79,80]. Contreras-Aguilar et al. [67] evaluated 21 salivary analytes in lame cows before and after hoof trimming and found that total esterase activity was higher in lame cows than in healthy cows and positively correlated with both numerical rating score and lesion score, suggesting potential value as a biomarker of lameness-associated physiological change. Similarly, Andjelić et al. [79] evaluated blood and milk metabolic parameters in 100 Holstein dairy cows and found that blood and milk metabolite patterns were associated with lactation stage and metabolic status, supporting the broader use of milk and blood measures for dairy cow health monitoring. Girdauskaitė et al. [80] further evaluated 91 early-lactation Holstein cows and found that lactate dehydrogenase activity was strongly associated with aspartate aminotransferase activity, while cows with higher lactate dehydrogenase tended to show lower rumination time and altered milk-monitoring indicators [80]. These findings emphasize the potential value of combining biochemical markers with production and behavioral data when evaluating early-lactation cow health [80].

Although physiological and behavioral indicators may improve sensitivity, particularly for early-stage or subtle cases, they are unlikely to replace locomotion-based detection. Instead, these measures are most useful as complementary inputs within multimodal systems, where behavioral, production, and physiological data can be interpreted alongside gait-based indicators to support more comprehensive lameness detection and production-level decision-making [6,33,45].

#### 3.3.4. Technological and Implementation Challenges

Multimodal ALD systems introduce several technical challenges related to data integration, including preprocessing, synchronization, and feature standardization across heterogeneous data streams [19,25]. High computational demands, infrastructure complexity, and installation costs may further limit scalability, while the lack of standardized data fusion methodologies and benchmarking frameworks complicates comparisons across studies [6,11,12,22,23,45,83]. Table 2 provides a comparative summary of performance metrics across ALD modalities, highlighting variability in accuracy, AUC, and study design among sensor-based, vision-based, and multimodal approaches. Reliance on internal validation rather than external multi-farm datasets may also overestimate system performance, thereby limiting confidence in real-world applicability [6,12,23,84]. Together with the absence of shared datasets, these inconsistencies restrict comparability across studies and delay consensus on optimal detection approaches [6,12,83]. This variability in reported performance metrics, summarized in Table 2, reinforces the need for unified evaluation frameworks.

Economic and infrastructure considerations also represent major barriers to adoption. Costs associated with hardware, installation, data storage, maintenance, and system integration remain substantial, particularly for small- and medium-scale operations [6,11,23]. Beyond the initial investment, ongoing expenses related to software updates, sensor replacement, and data management may further limit long-term feasibility [6,23]. Uncertainty surrounding return on investment also continues to restrict adoption, especially in the absence of standardized economic evaluations and long-term performance data [6,85].

**Table 2:** Comparative performance of automated lameness detection technologies in dairy cattle.

Detection Technology	Detection Type	Studies Reporting Conventional Accuracy (*n*)	Reported Accuracy Range (%)	Studies Reporting AUC (*n*)	Reported AUC Range	Contributing Studies
Wearable sensors/accelerometers	Behavioral and kinematic	3	76.0–87.0	0	N/A	[29,36,78]
Pressure/force platforms	Kinetic/weight distribution	0	N/A	2	0.71–0.88	[17,40]
2D computer vision/gait modeling	Direct gait/AI-based	11	80.1–100	0	N/A	[26,49,52,53,54,56,57,59,69,76,86]
3D computer vision/kinematic analysis	Direct gait/depth imaging	3	95.7–96.0	1	0.702–0.719	Accuracy: [58,60,61]; AUC: [38]
Infrared thermography	Thermal/lesion-associated screening	0	N/A	0	N/A	[18,62,63,64,65]
Multimodal/integrated sensing	Behavioral, physiological, production, and/or locomotion data	4	75.0–99.55	1	0.89	[33,41,70,71]

Notes: Values represent a descriptive synthesis of directly reported study-level performance and are not pooled or weighted meta-analytic estimates. The number of contributing studies is metric-specific; therefore, the number of studies contributing accuracy estimates may differ from the number contributing AUC estimates. Conventional classification accuracy was included only when explicitly reported by the original study and was not calculated from sensitivity and specificity. Balanced accuracy and other alternative classification metrics were not treated as conventional accuracy. AUC values were included only when directly reported and were not inferred from accuracy, sensitivity, specificity, or other performance measures. When a study reported multiple classification tasks, the value corresponding most directly to individual-cow lameness detection was used where clearly identifiable. Threshold-only results and metrics describing broader disease classification or herd-level lameness risk were retained in Supplementary Table S1 but were not incorporated into the ranges shown here.

Reliable internet connectivity represents an additional constraint for deployment of many modern ALD systems, particularly those that rely on cloud-based processing and real-time analytics [6,11,86]. Limited broadband access in rural regions may restrict data transmission, system integration, and timely decision-making, thereby reducing the practical utility of these technologies in commercial settings [86]. As shown in Table 3, commercially available systems vary substantially in cost, infrastructure requirements, and readiness for deployment, further influencing adoption across on-farm systems. Beyond technical and economic factors, successful implementation depends on usability and user acceptance [23,28,87,88]. High false alert rates may contribute to alarm fatigue and reduce trust in automated systems [23,28,87,88,89]. Effective use also requires integration within existing herd management workflows and sufficient technical literacy, which may vary across operations [6,88,90]. Consequently, adoption is influenced not only by diagnostic performance but also by system transparency, usability, and the ability to generate actionable insights that support production-level decision-making [6,11].

### 3.4. Application for Production

The successful implementation of ALD systems depends not only on diagnostic performance under controlled conditions but, more critically, on their reliability and usability within commercial production environments [11,12,23]. Although many technologies demonstrate high accuracy in experimental settings, their effectiveness often declines under real-world conditions due to environmental variability, infrastructure limitations, and integration challenges [6,11,12,23]. Within PLF, the value of ALD systems is therefore defined by their ability to integrate with existing management systems and support actionable decision-making [6]. This section focuses on how ALD technologies can be applied on dairy farms, highlighting practical factors that affect implementation, including environmental conditions, integration with existing farm systems, and insights from related livestock applications.

#### 3.4.1. Importance and Benefits of On-Farm Automated Lameness Detection Implementation

Within commercial dairy systems, ALD technologies are increasingly recognized for their potential to improve animal welfare, enhance production efficiency, and support data-driven management decisions through continuous monitoring and early detection [6,11,12]. Early identification of lameness is particularly important, as timely intervention has been associated with improved treatment outcomes, reduced lesion severity, and decreased duration of pain, while also mitigating negative impacts on milk production, reproductive performance, and culling risk [1,5,85,91,92]. In addition to welfare and production benefits, automated systems provide objective and standardized measurements that reduce observer variability and enable consistent longitudinal monitoring across herds [6,12,24]. However, the extent to which these benefits are realized depends on system accuracy, validation, and effective integration within production management practices [6,11].

#### 3.4.2. Environmental and On-Farm Challenges

Environmental variability remains a key barrier to on-farm implementation of ALD systems, contributing to reduced reliability and a gap between performance reported in controlled studies and commercial conditions [6,11,12,23]. This discrepancy has important implications for system validation and deployment, as technologies must demonstrate consistent performance across diverse production environments and management systems to be practically useful [6,11,12,22,23]. Addressing environmental robustness and ensuring multi-farm generalizability therefore remain critical for large-scale adoption of ALD technologies [6,12,22].

#### 3.4.3. Integration Within Precision Livestock Farming Systems

**Figure 3:**
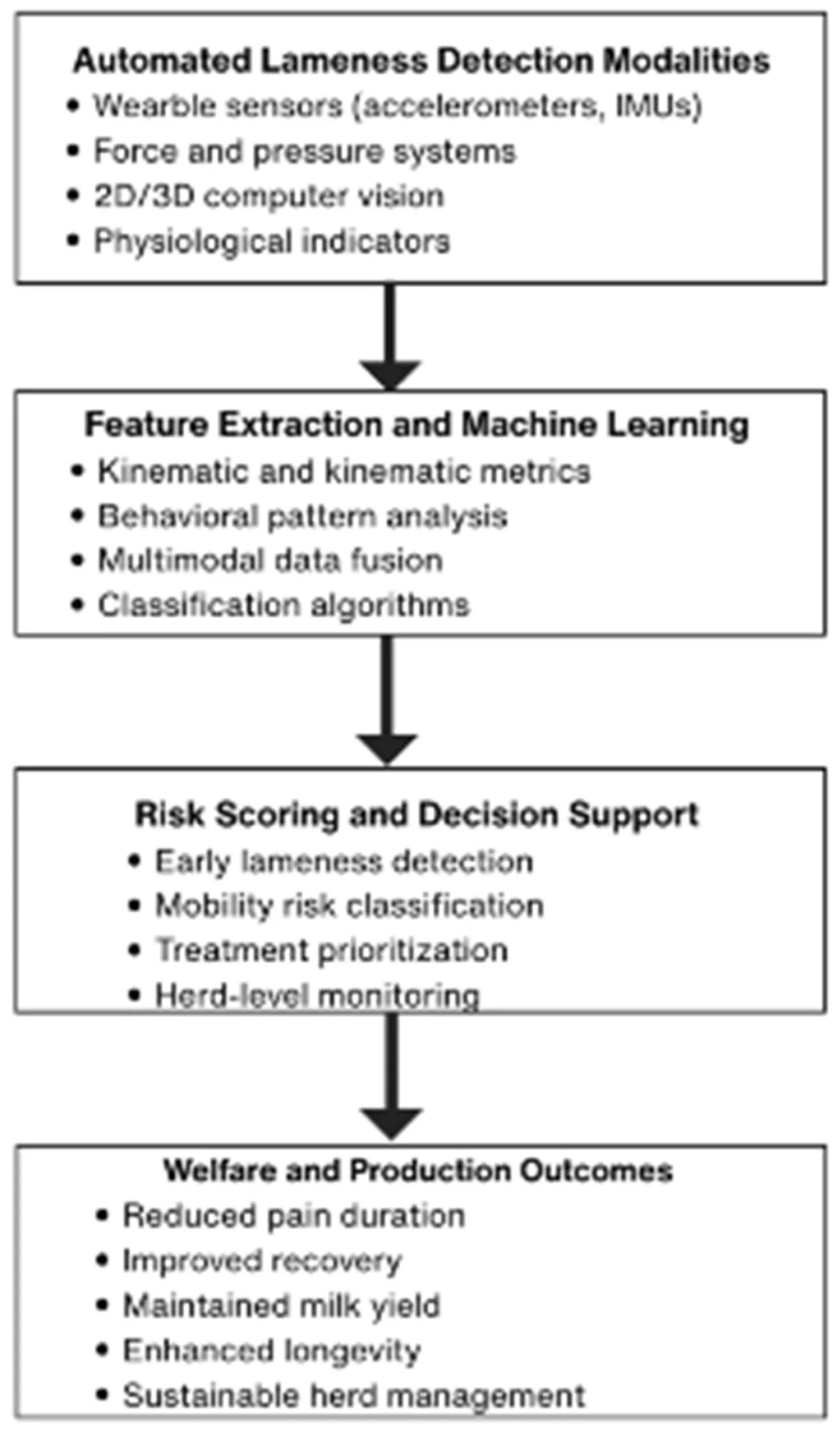
Conceptual framework of automated lameness detection (ALD) within precision livestock farming (PLF) systems. Multiple sensing modalities, including wearable sensors, force and pressure platforms, computer vision systems, and physiological indicators, feed into feature extraction and machine learning pipelines to generate mobility risk scores and clinical decision-support outputs. These outputs contribute to improved welfare, productivity, and data-driven herd management. The conceptual structure is based on previously described PLF and automated lameness detection frameworks [6,11,12]. Figure created by the authors in Procreate. Software was selected for figure-specific functionality.

Advanced ALD systems are increasingly integrated within PLF frameworks, where their value extends beyond standalone detection to continuous monitoring and decision support [6,11,27]. By linking locomotion data with behavioral, physiological, and production metrics, these systems enable real-time monitoring and support more proactive herd management strategies [6,24,33,39,45,93]. A conceptual representation of this integrated ALD-PLF data flow framework is presented in Figure 3, while Table 3 summarizes commercially available ALD systems, highlighting differences in sensing modality, validation status, cost, and commercial readiness. However, effective implementation depends on seamless integration with existing production management systems [6,11,27]. Challenges related to data interoperability, platform compatibility, and lack of standardized data structures can limit integration across technologies [6,11,12,22]. In addition, the increasing volume and complexity of multimodal data may reduce practical utility if not translated into clear, actionable outputs for production-level decision-making [6,19,25]. Addressing these limitations through improved data integration frameworks, user-centered system design, and decision-support tools will be critical to realizing the full potential of ALD technologies within PLF systems [6,11,90].

#### 3.4.4. Cross-Species Insights to Advance Dairy Lameness Detection

Although ALD systems have been most extensively developed in dairy cattle, research across other livestock species and equine medicine provides important opportunities to inform future technological development in dairy systems [6,12,94]. These cross-species advancements highlight emerging methodologies that may enhance detection accuracy, scalability, and integration within PLF frameworks [6,11]. Dairy cattle remain the most intensively studied species due to the substantial economic burden of lameness, the presence of structured walkways that facilitate sensor and camera deployment, and the long-standing use of standardized locomotion scoring systems [12,27,85]. Commercial implementations include wearable accelerometers, pressure-sensitive walkways, and camera-based gait analysis platforms deployed on-farm, particularly in Europe and North America [6,11,27]. As a result, dairy systems serve as the primary model for technological development and validation in automated locomotion monitoring [6,11,12,27]. However, further advancement of ALD systems in dairy cattle may benefit from the adoption and adaptation of technologies developed in other species [6,12,94].

In swine production, lameness contributes to premature culling in breeding sows and is associated with reduced reproductive performance and shortened productive lifespan [95,96]. Objective motion analysis systems, including infrared-based 3D imaging and automated gait monitoring technologies, have been used to quantify gait alterations and postural instability in pigs [95,96,97]. Force plates and pressure-sensitive flooring have also been applied to evaluate locomotor deficits associated with housing conditions, flooring type, and claw health [95,96,97]. However, compared to dairy systems, commercial deployment remains less advanced, and adoption is still emerging despite increasing interest driven by group housing systems [6,95]. These approaches may benefit dairy systems by informing the development of monitoring technologies capable of accounting for variability in housing design and flooring conditions, improving detection of subtle gait alterations in group-housed environments, and supporting more robust, real-time lameness detection under commercial conditions [6,95,97].

Additionally, equine medicine represents one of the most technologically advanced areas of veterinary gait analysis, with extensive use of force plates, IMUs, and high-speed motion capture systems in both clinical and research settings [94,98]. Wearable IMU networks and computer vision approaches enable detailed kinetic and kinematic assessment and are increasingly validated for field use [94,98,99]. However, these systems are typically designed for individual-level diagnosis rather than herd-level surveillance, limiting direct translation to large-scale livestock systems despite providing important methodological advancements [6,94,99]. Nevertheless, equine-based technologies provide a valuable foundation for advancing ALD systems in dairy cattle [6,94]. In particular, high-resolution kinematic analysis and wearable sensor networks developed in equine research may be adapted to improve detection sensitivity, refine gait asymmetry metrics, and enhance early identification of subtle locomotor abnormalities in dairy systems [6,94,99]. Translating these approaches to herd-level applications represents a key area for future research, particularly in developing scalable, automated monitoring systems suitable for commercial dairy environments [6,94,99].

Further, emerging research in small ruminants, including sheep and goats, suggests that wearable accelerometers, global positioning system (GPS) tracking, and thermal imaging may enable automated detection of gait deviations in extensive or pasture-based systems where routine visual observation is limited [21,100]. Accelerometry-based activity monitoring has been investigated as a tool for identifying locomotion abnormalities and behavioral changes associated with pain or injury [21]. However, commercial systems remain limited, and further validation is required before widespread implementation [6,12,21,100]. These approaches may benefit dairy systems by informing the development of monitoring technologies suited to pasture-based or hybrid production systems, where continuous visual assessment is not feasible [21,87,93,100]. In particular, the integration of wearable sensors and GPS-based tracking may enable continuous, remote monitoring of locomotion and behavior, improving detection of lameness in less-controlled environments and expanding the applicability of ALD systems beyond confined housing conditions [6,21,100].

Collectively, these cross-species applications demonstrate that automated locomotion monitoring is evolving from a species-specific innovation into a broader welfare assessment strategy within PLF systems [6]. Advances in dairy cattle research have established technical standards and validation frameworks, while innovations in other species provide complementary methodologies, such as high-resolution kinematic analysis, advanced imaging systems, and wearable sensor networks, that may accelerate the development of more sensitive and scalable lameness detection systems in dairy cattle [12,92,93,99,101]. Integrating these cross-species insights into dairy systems represents a key direction for future research, particularly for improving detection sensitivity, expanding monitoring across diverse farm environments, and enhancing system scalability within PLF frameworks [6,21].

## 4. Challenges and Future Perspectives

Despite substantial advances in ALD technologies, several technical and practical challenges continue to limit their widespread implementation in commercial dairy systems [6,11,12]. Variability in sensor performance, data quality, environmental conditions, and validation approaches contributes to inconsistent system accuracy and reliability across studies, highlighting a persistent gap between technological development and practical implementation [6,11,12,23]. In addition, infrastructure requirements, economic feasibility, and integration within existing herd management practices remain key determinants of adoption [6,11]. The following section outlines key limitations identified in the current literature and highlights priority areas for future research aimed at improving the development, validation, and real-world deployment of ALD systems.

### Future Directions and Research Needs

Although ALD technologies have advanced substantially, several research priorities remain essential to support large-scale implementation and long-term reliability [6,11,12]. A primary need is greater standardization of validation protocols. Variation in locomotion scoring systems, lameness thresholds, lesion confirmation methods, and performance metrics limits comparability across studies [6,12,22]. Development of standardized benchmarking frameworks, including consistent reporting of sensitivity, specificity, AUC, and external validation outcomes, would facilitate objective comparison and accelerate technological refinement [6,12,83].

External validation across diverse on-farm environments remains another critical priority. Many systems are evaluated under controlled conditions or single-farm datasets, which may not reflect variability in commercial operations [6,11,23,84]. Multi-farm and multi-region studies incorporating different housing systems, herd sizes, flooring types, and climatic conditions are needed to assess real-world robustness and generalizability [12,84]. This is particularly important for machine learning approaches, where performance may decline when models are applied outside their training environment [19,25,26,84]. Future work should also prioritize early-stage and subclinical lameness detection, as subtle locomotor changes remain more difficult to identify reliably [3,23,45,46,101,102,103].

Integration and data fusion represent promising avenues for improving diagnostic performance [6,19,24,33,70,72]. Multimodal systems combining kinematic, kinetic, behavioral, and physiological signals may enhance robustness by capturing multiple dimensions of locomotion and health simultaneously [6,19,24,33,70,72]. However, standardized data integration pipelines and transparent model architectures are needed to reduce overfitting and improve interpretability [19,25]. Greater emphasis on explainable AI may further support producer trust and practical adoption [6,25]. Moreover, economic evaluation and implementation research remain underdeveloped [6,28,85,88,104]. Longitudinal studies quantifying return on investment, labor savings, reduced culling rates, and production outcomes would strengthen the case for adoption in commercial systems [1,6,85]. In addition, improvements in user interface design, alert optimization, and integration with herd management software will be essential to maximize usability [6,11,90]. As these technologies mature, alignment with broader animal welfare and sustainability frameworks will be increasingly important, positioning ALD within integrated PLF systems [6,27,93,104].

## 5. Conclusions

Automated lameness detection technologies represent a significant advancement in PLF with strong potential to improve early detection and animal welfare in dairy systems. However, variability in farm environments, differences in validation approaches, and limitations in generalizability remain key barriers to consistent performance. Standardized evaluation frameworks and external validation across diverse farm settings will be essential to improve comparability and reliability. While these systems are best applied as decision-support tools rather than replacements for clinical assessment, continued advancements in AI and sensor integration are likely to enhance their practical utility. Successful implementation will ultimately depend on cost-effectiveness, ease of integration, and adoption within commercial dairy operations.

## Figures and Tables

**Table 1:** Relative detection capability of automated lameness detection modalities by lesion or presentation type.

System Type	Digital Dermatitis	Sole Ulcers	White Line Disease	Toe Lesions	General Gait Asymmetry	Behavioral Indicators
Wearable Sensors	Limited	Moderate	Moderate	Limited	High	High
Force/Pressure Systems	High	High	High	Moderate	Very High	Limited
Two-dimensional (2D) Vision Systems	Limited	Moderate	Moderate	Limited	High	Moderate
Three-dimensional (3D) Vision Systems	Limited	High	High	Moderate	Very High	Moderate
Infrared Thermography (IRT)	High	Moderate	Limited	Limited	Limited	Limited
Multimodal Systems	High	Very High	Very High	High	Very High	High

Notes: Qualitative categories (Limited, Moderate, High, Very High) reflect relative detection capability trends reported across included validation studies and are not pooled in quantitative estimates. These qualitative rankings are based on synthesis validation studies across pressure-based, wearable, and vision-based lameness detection systems [3,12,17,18,24].

**Table 3:** Overview of commercially available automated lameness detection (ALD) systems and their validation status in dairy cattle.

Technology Category	Technology Type	Key Features	Validation Evidence	Cost Level	Commercial Readiness	Sources
Depth-camera systems	Three-dimensional (3D) imaging	Spine curvature, body posture, kinematic analysis	Reported moderate-to-high performance, often in controlled settings	$$$	Research/limited commercial use	[1,15,72,90,91]
Ear-tag wearable systems	Wearable sensor	Activity, rumination, temperature, behavioral trends	Reported moderate-to-high classification performance	$$	Commercially available	[74,92,93]
Force plate systems	Force-based platform	Weight distribution, limb-loading asymmetry	Reported high performance under controlled or semi-controlled conditions	$$$$	Research and advanced applications	[23,67,92,93]
Vision-based gait analysis systems	Two-dimensional (2D) camera with artificial intelligence (AI)-based gait analysis	Posture and gait assessment; no wearable required	Reported moderate-to-high classification performance	$$	Commercially available/emerging	[41,58,72,73,86,87]
Leg-mounted accelerometer systems	Wearable motion sensor	Lying/standing time, step count, stride characteristics	Reported variable-to-moderate classification performance	$	Commercially available	[74,92,93]
Multimodal Precision livestock farming (PLF)platforms	Sensor, video, and/or force-data integration	Multisource data fusion; combines behavioral and locomotion indicators	Often reports high classification performance, but external validation remains limited	$$$$	Research prototype	[23,33,70,77,94]
Neck-mounted accelerometer systems	Wearable activity monitor	Activity, feeding behavior, behavioral monitoring	Reported moderate detection performance	$	Commercially available	[74,92,93,95]
Overhead camera tracking systems	Camera-based monitoring	Location tracking, posture, locomotion behavior	Reported moderate classification performance	$$$	Commercially available/emerging	[75,92,93]
Pressure mat walkway systems	Force/pressure-based platform	Force distribution, pressure loading, gait asymmetry	Reported high diagnostic performance under controlled conditions	$$$$	Research and limited commercial use	[75,92,93]

Notes: Cost level is presented qualitatively ($–) to reflect relative system expense, where $ indicates lower-cost wearable or single-sensor systems, $$ indicates moderate-cost systems such as multi-sensor or basic camera systems, $$$ indicates higher-cost systems requiring advanced imaging or infrastructure, and $$$$ indicates high-cost platforms involving specialized equipment or integrated monitoring systems. Values are derived from reported study-level performance metrics in the cited literature. Where direct accuracy was not reported, midpoint accuracy was calculated from sensitivity and specificity. Reported values represent descriptive synthesis rather than formal meta-analytic estimates due to variation in datasets, locomotion scoring thresholds, and validation methodologies across studies.

## Data Availability

No new data was generated or analyzed in support of this research. This article is a review of previously published studies, and all data supporting the findings are available within the cited literature.

## Supplementary Materials

The following supporting information can be downloaded at: https://www.mdpi.com/article/10.3390/ani16172643/s1.

## Abbreviations

The following abbreviations are used in this manuscript:

2D	Two dimensional
3D	Three dimensional
AI	Artificial intelligence
ALD	Automated lameness detection
AUC	Area under the curve
CNN	Convolutional neural network
GPS	Global positioning system
IMU	Inertial measurement units
IRT	Infrared thermography
PLF	Precision livestock farming
