# Supplementary material for: Hooves, Sensors, and Signals: Precision Approaches to Automated Lameness Detection in Dairy Cattle
Source: Animals (Basel). 2026 Aug 24;16(17):2643. doi: 10.3390/ani16172643 (PMC13564334; doi:10.3390/ani16172643)
Supplement: Supplementary file 1 [file animals-16-02643-s001.zip › animals-4495616-supplementary.pdf]

Supplementary Table S1. Studies included in the present review.

Manuscript Ref.	Study	Study type	Technology / modality	Validation context / sample	Accuracy (%)	AUC	Other reported metrics
58	Abdul Jabbar et al. (2017)	Primary validation	3D computer vision / depth video	22 Holstein Friesian dairy cows; overhead 3D depth camera; binary LS classification	95.7%	NR	Sensitivity = 100%; specificity = 75%
18	Alsaaod & Büscher (2012)	Primary validation	IRT	24 dairy cows; 626 observations; hind-claw lesion detection	NR	NR	Pre-trimming sensitivity = 85.7%, specificity = 55.9%; post-trimming sensitivity = 80.0%, specificity = 82.9%
29	Alsaaod et al. (2012)	Primary validation	Pedometric activity + lying behavior	Electronic behavioral monitoring	76%	NR	NR
62	Álvarez et al. (2025)	Primary validation	IRT	368 dairy cows; thermal images of hind feet	NR	NR	Higher coronary-band temperatures associated with higher LS and hoof pathology
43	Anagnostopoulos et al. (2023)	Primary validation	Computer vision / video	6,040 mobility scores from 3 farms	NR	NR	Sensitivity = 52%; specificity = 81%; agreement >80%
56	Barney et al. (2023)	Primary validation	Deep learning / pose estimation	25 video sequences containing 250 cows; threefold CV	100% binary; 94% severity	NR	κ = 0.8782; precision = 0.8650; recall = 0.9209
65	Bobić et al. (2025)	Review article	IRT	Review of IRT applications	NR	NR	Synthesized sensitivity, specificity, thresholds, and environmental effects
61	Bradtmueller et al. (2025)	Primary validation	3D kinematics + ML	3D gait coordinates + LSTM	96% (SD = 3%)	NR	Precision, recall, F1 = 0.96 ± 0.03
66	Bumbálek et al. (2026)	Primary validation	CV + IRT	160 Holstein cows; 14,322 infrared images	NR	NR	Hoof-detection mAP@50 ≈ 0.97

40	Chapinal et al. (2010)	Primary validation	Force/weight distribution + behavior	57 lactating cows	NR	0.71	Rear-leg weight variability strongest predictor
51	Czirok et al. (2026)	Primary validation	Computer vision	699 cows from 3 farms	NR	NR	Object-detection mAP@0.5 = 73.2%; body-part detection = 64.1-78.6%
36	Dai et al. (2025)	Primary validation	Wearable IMU	Early/severe lameness classification	80.6%	NR	Recall = 89.11%; F1 = 88.93%
44	de Mol et al. (2013)	Primary validation	Behavioral sensing	Same-farm development and validation	NR	NR	Sensitivity = 85.5%; specificity = 88.8%
91	Dervić et al. (2024)	Primary validation	Machine learning	6-farm development; 44-farm additional evaluation	NR	NR	Balanced accuracy = 79%; precision up to 93%
70	Dhaliwal et al. (2025)	Primary validation	Multimodal AI	6 Holstein cows over 21 d	99.55%	NR	Multimodal model outperformed single-modality models
41	Džermeikaitė et al. (2025)	Primary validation	Multimodal ML	272 early-lactation cows	97.04%	NR	Specificity = 100%; normalized MCC = 0.94
53	Jiang et al. (2022)	Primary validation	Computer vision	567 training and 243 validation/testing videos	96.61%	NR	Back-curvature model
69	Kang et al. (2025a)	Primary validation	Behavior + gait	Multiple gait/posture features	84%	NR	Macro-F1 = 0.81
76	Kang et al. (2025b)	Primary validation	Gait features + attention model	175 cows; 3,150 feature maps	92.80% severity; 99.05% binary	NR	Severity sensitivity = 89.21%, specificity = 94.60%; binary sensitivity = 100%, specificity = 98.57%
26	Kang et al. (2020)	Primary validation	Computer vision	100 naturally walking cows	96%	NR	Lame-hoof detection = 93%; correlation = 0.864
71	Lemmens et al. (2023)	Primary validation	AMS + sensor data / ML	10 farms; 594 animals	75%	NR	Sensitivity = 72%; specificity = 78%

86	Li et al. (2023)	Primary validation	Computer vision / temporal model	888 videos	98.89%	NR	Temporal micromotion model
31	Mazrier et al. (2006)	Primary validation	Pedometer	Activity reduction alerts	NR	NR	21/38 lame cows showed reduced activity 7-10 d before signs
77	Miekley et al. (2013)	Primary validation	PCA / multivariate	315 cows	NR	NR	Sensitivity = 73.8-87.8%; specificity = 54.8-61.9%
72	Mondini et al. (2026)	Primary validation	AMS + BCS + ML	323 cows; 3 farms	NR	NR	Balanced accuracy = 92%
54	Narli et al. (2025)	Primary validation	Computer vision	260 cows; back-keypoint analysis	97%	NR	Middle and caudal back regions most informative
25	Neupane et al. (2024)	Primary validation	Machine learning	310 cows over 4 months	>90% hoof-treatment; >85% severity	>0.74; >0.68	Values reported as thresholds
17	Pastell et al. (2010)	Primary validation	Force/weight distribution	61 cows; leg-weight ratio	NR	0.71-0.88	AUC = 0.87 for sole-ulcer vs. sound cows
42	Paulauskaitė-Tarasevičienė et al. (2026)	Broad health validation	Multimodal sensing + AI	88 cows; combined sick classification	91.62%*	0.94*	Not lameness-specific
59	Russello et al. (2024)	Primary validation	Video + pose estimation	9 keypoints; 6 locomotion traits	80.1%	NR	76.6% with 1 trait; 79.9% with 3; 80.1% with 6
57	Russello et al. (2026)	Primary validation	Pose estimation + BiLSTM	Binary lame/non-lame classification	85%	NR	Feature-based approach = 80%
63	Spadola et al. (2026)	Primary validation	Clinical + IRT	15 cows; hoof-disease vs. controls	NR	NR	Thermal differences associated with hoof pathology
64	Stokes et al. (2012)	Primary validation	IRT	Foot-lesion screening	NR	NR	27°C threshold: sensitivity = 80%; specificity = 73%
84	Swartz et al. (2026)	Primary validation	Camera + predictive model	Development and prospective validation	NR	NR	Best F0.5 = 59.1; test PPV = 92.3%; prospective PPV = 13.7%

48	Swartz et al. (2024)	Primary validation	Camera-based detection	3 farms; trimming-event comparisons	NR	NR	Higher locomotion scores preceded lesion-associated trimming
49	Szyc et al. (2025)	Primary validation	Video-based detection	832 cows	82.1%	NR	Expert-comparison accuracy = 87.2%
55	Taghavi et al. (2024)	Primary validation	Deep-learning keypoints	34 cows; 758 paired-frame samples	NR	NR	PCKh0.2 = 89% overall; back = 59%
78	Taneja et al. (2020)	Primary validation	ML + fog computing	Commercial farm; 150 cows	87%	NR	Detected lameness ≈3 d before farmer
60	Tun et al. (2026)	Primary validation	Depth imaging + DL	Unseen test data	95.95%	NR	F1 = 96.06%; accuracy CI = 94.8–97.1%
33	Van Hertem et al. (2013)	Primary validation	Milk yield + rumination + neck activity	118 cows; 10-fold CV	81% CV; 86% complete-data model	0.89	CV sensitivity = 77%, specificity = 84%; complete-data sensitivity = 89%, specificity = 85%
38	Van Hertem et al. (2018)	Primary validation	3D computer vision	633 milking sessions	NR	0.702-0.719	AUC = 0.719 back curvature; 0.702 back posture
90	Warner et al. (2020)	Herd-level validation	Production records + ML	229 dairy herds	NR	0.73-0.75; stacked = 0.76	Herd-level rather than individual-cow prediction
52	Zhao et al. (2023)	Primary validation	Computer vision + ML	212 walking videos; 10-fold CV	89.2%	NR	Recall = 90.7%
